# Does payment for performance increase performance inequalities across health providers? A case study of Tanzania

**DOI:** 10.1093/heapol/czy084

**Published:** 2018-10-31

**Authors:** Peter Binyaruka, Bjarne Robberstad, Gaute Torsvik, Josephine Borghi

**Affiliations:** 1 Centre for International Health, University of Bergen, Bergen, Norway; 2 Department of Health System, Impact Evaluation, and Policy, Ifakara Health Institute, Dar es Salaam, Tanzania; 3 Department of Global Health and Development, Chr. Michelsen Institute, Bergen, Norway; 4 Department of Economics, University of Oslo, Oslo, Norway; 5 Department of Global Health and Development, London School of Hygiene & Tropical Medicine, 15-17 Tavistock Place, London, UK

**Keywords:** Payment-for-performance, inequality, impact evaluation, incentive design, Tanzania

## Abstract

The impact of payment-for-performance (P4P) schemes in the health sector has been documented, but there has been little attention to the distributional effects of P4P across health facilities. We examined the distribution of P4P payouts over time and assessed whether increased service coverage due to P4P differed across facilities in Tanzania. We used two service outcomes that improved due to P4P [facility-based deliveries and provision of antimalarials during antenatal care (ANC)], to also assess whether incentive design matters for performance inequalities. We used data from 150 facilities from intervention and comparison areas in January 2012 and 13 months later. Our primary data were gathered through facility survey and household survey, while data on performance payouts were obtained from the programme administrator. Descriptive inequality measures were used to examine the distribution of payouts across facility subgroups. Difference-in-differences regression analyses were used to identify P4P differential effects on the two service coverage outcomes across facility subgroups. We found that performance payouts were initially higher among higher-level facilities (hospitals and health centres) compared with dispensaries, among facilities with more medical commodities and among facilities serving wealthier populations, but these inequalities declined over time. P4P had greater effects on coverage of institutional deliveries among facilities with low baseline performance, serving middle wealth populations and located in rural areas. P4P effects on antimalarials provision during ANC was similar across facilities. Performance inequalities were influenced by the design of incentives and a range of facility characteristics; however, the nature of the service being targeted is also likely to have affected provider response. Further research is needed to examine in more detail the effects of incentive design on outcomes and researchers should be encouraged to report on design aspects in their evaluations of P4P and systematically monitor and report subgroup effects across providers.

**Key Messages**

- Inequality in payouts favoured better-off facilities, but declined over time.
- Lower baseline performers improved most on institutional deliveries coverage.
- Rural and middle wealth facilities improved most on deliveries coverage.
- Performance on antimalarial provision was similar across facilities.

## Introduction

Payment-for-Performance (P4P) programmes, involving financial incentives (payouts) to healthcare workers and healthcare facility for achievement of pre-defined performance outcomes, are aimed at improving the quality of care and, especially in low- and middle-income countries (LMICs) are aimed to increase service coverage and strengthen health systems more generally (Meessen *et al.*, 2011; Witter *et al.*, 2013). The measured effects of P4P on healthcare coverage and quality are mixed across programmes and settings (Gillam *et al.*, 2012; Witter *et al.*, 2012; Eijkenaar *et al.*, 2013; Das *et al.*, 2016; Renmans *et al.*, 2016; Mendelson *et al.*, 2017).

To date, most evaluations of P4P schemes have largely focused on average programme effects, and paid less attention to how this remuneration system affect the distribution of programme effects (Markovitz and Ryan, 2016; Sherry *et al.*, 2017). The heterogeneity of P4P effects on service use among populations have been documented in the literature (Alshamsan *et al.*, 2010; Renmans *et al.*, 2016; Van de Poel *et al.*, 2016; Binyaruka *et al.*, 2018). However, from a theoretical point of view, it is not clear how P4P will affect the distribution of performance/performance inequalities across service providers. P4P could give the facilities that are lagging behind extra motivation to catch up and it may be easier to increase performance from a low level (Alshamsan *et al.*, 2010; Meessen *et al.*, 2011; Fritsche *et al.*, 2014). But P4P could also increase performance inequalities by rewarding facilities that are better able to perform (e.g. better resourced facilities) (Ireland *et al.*, 2011). The distributional effects of P4P will also depend, for example, on the exact design of the incentive scheme, and whether the reward depend linearly or non-linearly on performance score (Mehrotra *et al.*, 2010; Van Herck *et al.*, 2010; Levitt *et al.*, 2012; Eijkenaar, 2013; Miller and Babiarz, 2013).

In this study, we measured how P4P in Tanzania affected service coverage and facility performance across facilities with different characteristics, and whether the design of performance incentives enhanced or mitigated inequalities in service provision across facilities. This assessment is important especially in LMICs given the substantial variation in health facility readiness to deliver services (MoHSW, 2013; O’Neill *et al.*, 2013).

### P4P intervention in Tanzania

The public sector dominates the Tanzanian health system, private for profit and the voluntary sector (faith-based) serve as important supplements (MoHSW, 2015). The public health system has a hierarchal administrative structure with three main facility levels of care: dispensaries, health centres and hospitals. Dispensaries and health centres provides primary healthcare services, and hospitals are referral facilities.

In 2011, the Ministry of Health and Social Welfare (MoHSW) in Tanzania, with support from the Government of Norway, introduced a P4P pilot scheme in all seven districts of Pwani region. Pwani region has >300 health facilities covering a population of just over a million (NBS, 2013). All facilities providing maternal and child health (MCH) services in Pwani were included in the scheme. The P4P scheme was introduced to reduce maternal, neonatal and child morbidity and mortality by improving the coverage and quality of MCH services. It also aimed to inform the national P4P roll out. P4P incentives were tied to coverage of services (e.g. facility-based/institutional delivery) and content of care targets [e.g. provision of Intermittent Preventive Treatment (IPT) doses for malaria during antenatal care (ANC)] (Borghi *et al.*, 2013; Binyaruka *et al.*, 2015). Since P4P aimed to increase service coverage, performance targets were set based on coverage rates. For example, facilities were rewarded with extra funding if facility-based deliveries surpassed a target percentage of all deliveries, and if the fraction of pregnant women that received at least two doses of IPT (IPT2) were above a target (Table 1).

**Table 1.** Service indicators and performance targets for facilities implementing P4P in Tanzania

P4P service indicators	Method	Baseline coverage (previous cycle)				
		0–20%	21–40%	41–70%	71–85%	85%+
**Coverage indicators**						
% of institutional/facility-based deliveries	Percentage point increase	15%	10%	5%	5%	Maintain
% of mothers attending a facility within 7 days of delivery.	Percentage point increase	15%	10%	5%	5%	Maintain
% of women using long term contraceptives	Percentage point increase	20%	15%	10%	Maintain above 71%	Maintain
% children under 1 year received measles vaccine	Overall result	50%	65%	75%	80%+	Maintain
% children under 1 year received Penta 3 vaccine	Overall result	50%	65%	75%	80%+	Maintain
% of complete partographs	Overall result	80%	80%	80%	80%+	Maintain above 80%
HMIS reports submitted to district managers on time and complete	Overall result	100%	100%	100%	100%	100%
**Content of care indicators**						
% ANC clients receiving two doses of IPT	Overall result	80%	80%	80%	80%+	Maintain above 80%
% HIV+ ANC clients on ART	Overall result	40%	60%	75%	75%+	Maintain
% of children receiving polio vaccine (OPV0) at birth	Overall result	60%	75%	80%	80%+	Maintain

Health managers were rewarded based on the overall performance of facilities in their district/region. Managers also had their own indicators that includes, maternal and newborn deaths audited properly and timely; reducing stock-out rates of essential drugs; timely reporting the facility data from district to regional level, and from regional to national level.

*Source*: The United Republic of Tanzania, Ministry of Health and Social Welfare. 2011. The Coast Region Pay for Performance (P4P) Pilot: Design Document.

85%+, 85% or more; 80%+, 80% or more; HMIS, Health Management Information System; ANC, antenatal care.

There were two methods of target setting (Table 1): a single threshold (absolute coverage target) and multiple thresholds based on baseline performance/previous cycle (relative change/overall result). For multiple thresholds, each group of facilities faced an absolute threshold based on baseline performance: Group 1 (0–20% coverage of said indicator), Group 2 (21–40%), Group 3 (41–70%), Group 4 (71–85%) and Group 5 (>85%). Group 5 was required to improve or maintain coverage for payment. District and regional managers were rewarded for the performance of facilities in their district or region.

Performance data were compiled by facilities and verified by the P4P implementing agency every 6 months (one cycle) before payments. The maximum payout per cycle differed by facility level of care: USD 820 per cycle for dispensaries; USD 3220 for health centres and USD 6790 for hospitals. From the total payout earned, the largest share (90% in hospitals and 75% in lower level facilities) was for staff bonuses, while the remainder was for facility improvement and to increase demand. P4P payments were additional to regular government funding for operational costs and salaries unrelated to performance. Full payment per indicator was made if 100% of a given target was achieved, 50% of payment was made for 75–99% achievement and no payment was made for lower levels of performance. Staff bonuses were almost equivalent to 10% of their monthly salary if all targets were fully attained. The maximum payout for district and regional managers was USD 3000 per cycle (Borghi *et al.*, 2013).

An impact evaluation of the P4P programme in Tanzania showed a significant positive effect on two out of the eight incentivized service indicators: facility-based delivery rate and provision of antimalarials during ANC (Binyaruka *et al.*, 2015). The programme also increased the availability of drugs and supplies, increased supportive supervision, reduced payment of user fees and resulted in greater provider kindness during delivery care (Binyaruka *et al.*, 2015; Anselmi *et al.*, 2017; Binyaruka and Borghi, 2017; Mayumana *et al.*, 2017).

### Conceptual framework

**Figure 1.**
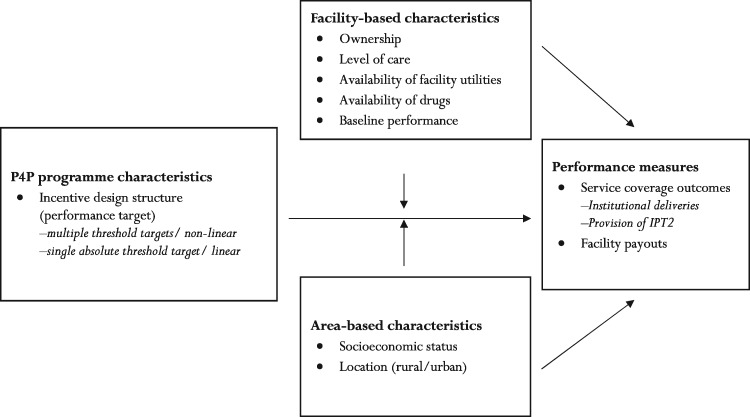
Conceptual framework for the determinants of performance in pay-for-performance programmes [we modified a conceptual framework which was initially developed by Rittenhouse et al. (2010) and Markovitz and Ryan (2016)]

To conceptualise the pathways to distributional effects of P4P among health providers, we adapted the theoretical framework by Rittenhouse *et al.* (2010) and Markovitz and Ryan (2016) to the Tanzanian context (Figure 1).

In an incentive system like the one implemented in a P4P pilot in Tanzania, with a hierarchy of performance targets, two factors play a role for how incentives affect the distribution of performance across facilities; the distance from current performance to the target and how costly it is for the facility to reach the target level. The costs of increasing performance depend both on effort costs and on enabling factors.

Suppose performance in period *t*($p_{t}$) is given by facility-level effort ($e_{t}$), and a set of structural/enabling factors ($x_{t}$): $p_{t}$ =*p*($e_{t}$, $x_{t}$). Performance is also assumed differentiable and weakly increasing in both arguments: $\frac{\partial p}{\partial e}$* *≥* *0, $\frac{\partial p}{\partial x}$ ≥0*.* We then consider two types of facilities: those with higher ($p_{0}^{H}$) and lower baseline performance ($p_{0}^{L}$). At baseline we have: $\Delta^{0}$= $p_{0}^{H}$ – $p_{0}^{L}$ > 0, and after P4P is introduced we have $\Delta^{1}\;$= $p_{1}^{H}$ – $p_{1}^{L}$. P4P incentive design structure and/or structural factors can affect performance across facilities over time, resulting in convergence in performance/positive distributional effects ($\Delta^{0}>\Delta^{1}$); divergence in performance/negative distributional effects ($\Delta^{0}<\Delta^{1}$); or similar performance across facilities (i.e. zero distributional effects) ($\Delta^{0}=\Delta^{1}$). We discuss the extent to which the incentive design (P4P target setting) and structural factors (facility- and area-based characteristics) affect performance across facilities.

#### Incentive design effect

We considered only target setting approach as potential incentive design element to affect performance (Figure 1). P4P schemes can reward using fee-for-service, geographical targeting, relative performance, single absolute threshold targets or multiple threshold targets (Rosenthal *et al.*, 2005; Rosenthal and Dudley, 2007; Mehrotra *et al.*, 2010; Eijkenaar, 2013; Fritsche *et al.*, 2014). The distributional effects of P4P schemes will partly depend on how incentives, and especially targets, are designed. We specifically focus on absolute and multiple thresholds target since these were used in the Tanzanian P4P scheme.

Multiple threshold target designs can enhance convergence in performance (Rosenthal *et al.*, 2005; Mehrotra *et al.*, 2010; Eijkenaar, 2013) because they account for baseline performance and provide incentives for lower performers to catch up. However, absence of systematic convergence in performance with this design has been observed in the UK (Sutton *et al.*, 2012). Absolute single threshold/linear targets can enhance divergence in performance if some providers are far above and below the target (Heath *et al.*, 1999; Rosenthal and Dudley, 2007; Mehrotra *et al.*, 2010; Mullen *et al.*, 2010; Miller and Babiarz, 2013). Improvement is most likely for providers/facilities that are close to achieve the threshold target. Top performers have no incentive to improve, and those far below the target may perceive it as unattainable, a phenomenon referred to as ‘goal-gradient’ theory (Heath *et al.*, 1999). A single target design fails to account for any variation in baseline performance (Rosenthal *et al.*, 2005; Mehrotra *et al.*, 2010; Mullen *et al.*, 2010; Eijkenaar, 2013).

#### Structural effect

Variation in facility- and area-based factors that are potentially responsible for inequalities in baseline performance can also affect overall facility performance over time (Figure 1) (Markovitz and Ryan, 2016). This is given by $\frac{\partial p}{\partial x}$*≥* 0. We further assume the change in effort devoted to affect performance $\frac{\partial p}{\partial e}$ is increasing in *x*, that is $\frac{\partial \frac{\partial p}{\partial x}\;}{\partial e}\;$> 0. If facilities invest initial bonus payments in enabling factors, this may improve their future performance, but general predictions of effects based on variation in structural factors are difficult to make (Markovitz and Ryan, 2016). We hypothesise that public facilities in Tanzania are better able to respond to incentives than non-public providers, as they can offer free MCH services (under the fee exemption policy) and have more financial autonomy (Mayumana *et al.*, 2017). However, it is also possible that P4P can level the playing field across providers of different ownership status (Meessen *et al.*, 2011). We further hypothesise that facilities with greater resource availability (e.g. essential drugs) are better able to increase patient demand than their counterparts (Donabedian, 1988; Alderman and Lavy, 1996; WHO, 2004); and that dispensaries are less able to respond to incentives compared with health centres and hospitals since they are more resource constrained (MoHSW 2013).

Regarding area-based factors, facilities with wealthier catchment populations may respond better to incentives, as they can more readily increase service use and revenue through user fees (Castro-Leal *et al.*, 2000; Victora *et al.*, 2000; Doran *et al.*, 2008; Chien *et al.*, 2012). Facilities in rural areas may be less able to respond to incentives than their urban counterparts, because of human resource shortages, poor road infrastructure, and more scattered and disadvantaged populations (Munga and Maestad, 2009; Witter *et al.*, 2013; Fritsche *et al.*, 2014).

Apart from the above hypothesized pathways (incentive design and structural effect), provider response may also depend on the nature of the services targeted or incentivized. This is because performance improvement can be harder for some services compared with other services and this may confound the initial hypothesises of incentive design and structural effect. For instance, less efforts are needed by providers to influence clients’ continuation of care than initiation of care (Gertler and Vermeersch, 2013).

## Materials and methods

### Study design and data sources

This study was part of the large impact evaluation of the P4P scheme in Pwani region (Borghi *et al.*, 2013; Binyaruka *et al.*, 2015). The P4P evaluation study surveyed all seven districts in Pwani region (intervention arm), and four districts from Morogoro and Lindi regions (comparison arm). Comparison districts were selected to be comparable to intervention districts in terms of poverty and literacy rates, the rate of institutional deliveries, infant mortality, population per health facility and the number of children under 1 year of age per capita (Borghi *et al.*, 2013).

Baseline data at facility and household-levels were collected in January 2012, with a follow-up round 13 months later. For each study arm, data on facility ownership (public or non-public facility), level of care (hospital, health centre or dispensary), availability of medical inputs (considered 37 essential drugs) and rural/urban location was obtained from 75 sampled facilities providing MCH services (6 hospitals, 16 health centres and 53 dispensaries). Data on socioeconomic status of the facility catchment populations and service coverage rates were obtained from households with women who had delivered in the 12 months prior to the baseline and endline surveys. We randomly sampled 20 eligible households from each facility’s catchment area, making a total of 1500 households in each arm per survey round. Facility payout data were obtained from the implementing agency for all incentivized indicators for the 75 intervention facilities in our sample over seven payment cycles (2011–14).

### Performance outcomes

We considered two facility performance outcomes. First, for each facility in the intervention arm and for each of seven payment cycles, we generated a ‘payout score’. That score was constructed as the bonus payout received divided by the maximum potential payout (all targets had been met) and multiplied by 100. Payout score was used to capture for each level of care the relative facility performance. Second, we estimated facility-level average service coverage rates for households in the facility catchment area from both study arms. Our coverage rates were estimated using only two incentivized services which improved significantly on average due to P4P (Binyaruka *et al.*, 2015); that is, the coverage of facility-based deliveries and provision of two doses of IPT for malaria during ANC (referred to as IPT2). We therefore considered only these two service outcomes to assess whether P4P effect differed across facilities.

### Subgroups of facilities for distributional analyses

To examine whether incentive design and structural effects affected performance outcomes, we identified facility subgroups as shown in Table 2, pertaining to their baseline performance for the two incentivized indicators (above or below the median); facility characteristics (ownership, level of care, availability of utilities, rural-urban location); an un-weighted index of drug availability at baseline (Supplementary AppendixTable S1); and wealth status of the catchment population, based on mean wealth index scores across households in the facility-catchment area generated by principal component analysis (Vyas and Kumaranayake, 2006) (Supplementary AppendixTable S2).

**Table 2.** Baseline facility and area-based characteristics by study arms

Characteristics	Description	Intervention (*n* = 75)	Comparison (*n* = 75)	Difference (*P*-value)
**Panel A: facility-based characteristics**				
Facility ownership	=1 for public owned (%)	84.0	82.7	1.3 (0.828)
Facility level of care	=1 for dispensary (%)	70.7	70.7	0.0 (1.000)
Availability of facility utilities	=1 for electricity and water supply (%)	54.7	52.0	2.7 (0.745)
Availability of drugs—index	Mean index (0–1) of 37 drugs [SD]	0.61 [0.16]	0.66 [0.12]	**–0.05 (0.031)**
Availability of drugs—subgroup	=1 for availability below the median (%)	57.3	42.7	**14.6 (0.073)**
Baseline coverage level (deliveries)	=1 for facility below the median (%)	53.3	46.7	6.6 (0.418)
Baseline coverage level (IPT2)	=1 for facility below the median (%)	54.6	45.3	9.3 (0.256)
**Panel B: area-based characteristics**				
Wealth status index	Mean wealth index [SD]	–0.43 [1.8]	0.32 [2.4]	**–0.75 (0.028)**
Wealth status—tercile 1	=1 for poorest population (%)	40.0	26.7	**13.3 (0.084)**
Wealth status—tercile 2	=1 for middle wealth population (%)	34.7	32.0	2.7 (0.731)
Wealth status—tercile 3	=1 for least poor population (%)	25.3	41.3	**–16.0 (0.038)**
Facility location	=1 for facility in rural district (%)	78.7	84.0	–5.3 (0.405)

Three quantiles (terciles) were used for wealth status of the facility’s catchment population; Availability of drugs include 37 drugs and analysis used a dummy variable classified based on baseline availability distribution (=1 for availability below the median/bottom half and 0, otherwise); SD, standard deviation; reference category in brackets: public (vs non-public), dispensary (vs health centre and hospital), with electricity and water supply at baseline (vs none), baseline availability of drugs below the median/in bottom half (vs top half), baseline lower performer/below the median (vs higher performer), rural (vs urban district); for distributional analyses, wealth index and drugs availability index were re-classified on each arm separately and equally to avoid the imbalance across arms at baseline.

### Analysis

We first compared the sample means at baseline for each of the facility subgroups across study arms, and examined eventual differences between study arms using the *t*-test.

#### Distribution of bonus payouts

To assess how bonus payouts were distributed across intervention facilities, we used three measures of inequality: an absolute measure (the gap) and two relative measures [the ratio and the concentration index (CI)] (O'Donnell *et al.*, 2008; WHO, 2013). The gap was measured as the difference in payout scores between facility subgroups. The ratio was measured as the ratio of payout scores between subgroups. In relation to wealth subgroups, a positive (negative) gap and a ratio greater (less) than one defines a pro-rich (pro-poor) distribution, respectively. A gap of zero and a ratio of one defines an equal distribution. We tested whether the gaps were significantly different from zero by using *t*-tests.

The CI was computed on a ranking variable of area-based wealth status to examine wealth-related inequality in the distribution of payouts (Kakwani *et al.*, 1997; O'Donnell *et al.*, 2008). The CI ranges between [−1 and +1], whereby zero indicate equality between wealth subgroups, while negative and positive values indicate that payouts are pro-poor and pro-rich, respectively. We tested whether the CIs were significantly different from zero.

#### Heterogeneity in service coverage outcomes

We measured the difference in mean baseline coverage of the two incentivized services between facility subgroups (the coverage gap; WHO, 2013) and tested for significant differences between subgroups.

Based on the two incentivized services that were improved by P4P (i.e. facility-based deliveries by 8.2% points, and provision of IPT2 by 10.3% points) (Binyaruka *et al.*, 2015), we assessed whether the effects differed by facility subgroup. We used a linear difference-in-differences regression model with a three-way interaction term between the average treatment effect (${P4P}_{i}\times \delta_{t}$) and facility subgrouping variable $G_{i}$. The associated two-order interaction terms were also included in the model as shown in Equation (1).

(1) $$Y_{it}=\beta_{0}+\beta_{1}{(P4P}_{i}\times \delta_{t})+\beta_{2}\delta_{t}+\beta_{3}Z_{it}+\beta_{4}{(P4P}_{i}\times \delta_{t}\times G_{i})\;+{\beta_{5}{(G}_{i}\times \delta_{t})+\gamma }_{i}+\varepsilon_{it}$$

where $Y_{it}$ is the service coverage outcome of facility *i* at time *t*. ${P4P}_{i}$ is a dummy variable, taking the value 1 if a facility is exposed to P4P and zero otherwise. We controlled for unobserved time-invariant facility-level characteristics $\gamma_{i}$ with facility fixed-effects estimation, and included $\delta_{t}\;$for year fixed-effects. We also controlled for time-varying facility-level covariates $Z_{it}$ (availability of electricity and water supply, and the mean wealth index for households sampled in the catchment area of the facility) as potential confounding factors. The error term is $\varepsilon_{it}$. Our statistical inference for regression was based on standard errors clustered at the facility level to account for serial correlation of $\varepsilon_{it}$ at the facility level. The coefficient of interest for the differential effect across facility subgroups is $\beta_{4}$.

Causal inference using the difference-in-differences approach relies on the key identifying assumption that the trends in outcomes would have been parallel across study arms in the absence of the intervention (Khandker *et al.*, 2010). While this cannot be formally tested, we justified the assumption by verifying that the pre-intervention trends were parallel in Tanzania (Binyaruka *et al.*, 2015; Anselmi *et al.*, 2017). This was verified in women who had delivered in the past 12 months at baseline for the following outcomes for which we had monthly data: share of institutional deliveries, caesarean section deliveries, women who breastfeed within 1 h of birth, and women who paid for delivery care. We also verified pre-intervention trends to be parallel in facility service utilization levels based on patient registers.

We performed some robustness checks. First, we re-estimated the model for facility-based deliveries excluding hospitals (8% of facilities per arm), as hospitals have less clearly defined catchment populations. Second, we clustered the standard errors at the district level and used a bootstrapping method to adjust the small number of district-clusters (Cameron and Miller, 2015). Third, we reclassified the mean wealth scores into two quantiles (below or above the median) to check whether the wealth effect was sensitive to classification of the wealth groupings. Lastly, apart from using a conventional parametric test (a *t*-test) to assess whether differences in payouts between subgroups were significant, a non-parametric test (Wilcoxon rank-sum test) was also used (Kitchen, 2009). All the analyses were performed using STATA version 13.

## Results

Facility and area-based characteristics were generally similar in the intervention and comparison arms at baseline (Table 2), although intervention facilities served poorer populations, and had marginally lower availability of drugs than comparison facilities.

### Distribution of bonus payouts

There was an increase in average payout scores between payment cycle 1 (50.1% of total potential payout) and cycle 7 (77.7%) (Table 3), and the payouts were highest for facilities with least poor catchment populations. This pro-rich effect was confirmed by positive equity gaps and concentration indices, and an equity ratio that was greater than one across all payment cycles (Table 3, Columns 5–7). The inequalities were generally stronger in early compared with later cycles (Table 3).

**Table 3.** Distribution of facility payout scores by wealth status of the catchment populations

Payment cycle	All	Area-based wealth status (terciles)			Equity		**CI (***P***-value)**
	Mean [SD]	Least poor	Middle	Poorest	Gap (*P*-value)	Ratio	
	(1)	(2)	(3)	(4)	(5)	(6)	(7)
CYCLE 1 (%)	50.1 [19.4]	54.7	52.3	43.1	**11.6 (0.027)**	1.27	**0.042 (0.099)**
CYCLE 2 (%)	50.3 [19.1]	58.4	49.7	42.4	**16.0 (0.002)**	1.38	**0.088 (0.000)**
CYCLE 3 (%)	64.6 [18.8]	69.2	65.1	59.6	**9.6 (0.062)**	1.16	**0.036 (0.054)**
CYCLE 4 (%)	67.5 [19.5]	67.8	69.6	65.1	2.7 (0.623)	1.04	0.007 (0.699)
CYCLE 5 (%)	74.5 [18.5]	75.3	74.9	73.4	1.9 (0.707)	1.03	0.007 (0.669)
CYCLE 6 (%)	69.6 [20.1]	72.0	75.3	61.3	**10.7 (0.046)**	1.17	**0.035 (0.058)**
CYCLE 7 (%)	77.7 [16.3]	79.2	76.9	76.9	2.3 (0.619)	1.03	0.006 (0.672)
Pooled—all cycles (1–7) (%)	64.7 [11.7]	68.1	66.3	60.5	**7.6 (0.015)**	1.13	**0.027 (0.022)**

Analysis restricted to intervention facilities only (*n* = 75); *p*-values in Column (5) were from *t-*test of the null hypothesis that the gap [Columns (2)–(4)] is equal to zero; *p*-values in Column (7) were for testing the null hypothesis of zero CI; SD, standard deviation; terciles for wealth status were generated with equal-size from intervention arm separately; Gap, least poor—poorest; ratio, least poor/poorest; the results were generally similar in Column (5) when non-parametric test (Wilcoxon rank-sum) is used (Supplementary Table S6).

Facilities with greater availability of drugs at baseline, hospitals and health centres had significantly higher payout scores than facilities with more limited drug availability and dispensaries (Table 4). The equity ratios were ∼1, near equality, between most subgroups (Table 4).

**Table 4.** Distribution of facility payout scores by other subgroups of facilities

Facility subgroups	By payment cycle							Pooled average cycles
	Cycle 1	Cycle 2	Cycle 3	Cycle 4	Cycle 5	Cycle 6	Cycle 7	Cycles 1–7
Facility location								
Rural (%)	52.2	48.5	66.3	69.5	76.4	71.3	80.0	66.4
Urban (%)	42.3	56.7	58.3	60.1	68.1	63.2	68.9	59.7
Gap (%)	9.9**	–8.2*	8.0	9.4*	8.3	8.1	11.1**	6.7*
Ratio	1.2	0.9	1.1	1.2	1.1	1.1	1.2	1.1
Ownership status								
Public owned (%)	49.9	49.5	66.0	68.8	75.8	70.0	78.4	65.6
Non-public (%)	50.9	54.4	56.9	60.6	66.7	67.1	73.6	61.5
Gap (%)	–1.0	–4.9	9.1	8.2	9.1	2.9	4.8	4.1
Ratio	1.0	0.9	1.2	1.1	1.1	1.0	1.1	1.1
Level of care								
Dispensary (%)	47.7	46.9	60.2	63.5	71.5	66.9	75.4	61.9
HC and hospital (%)	55.8	58.3	75.3	77.0	81.7	75.8	82.9	72.4
Gap (%)	−8.1*	−11.4**	−15.1***	−13.5***	−10.2***	−8.9**	−7.5**	−10.5***
Ratio	0.9	0.8	0.8	0.8	0.9	0.9	0.9	0.9
Electricity and water supply								
Available (%)	53.6	51.9	66.7	69.1	76.8	71.3	81.1	67.2
None (%)	45.9	48.3	62.1	65.5	71.7	67.5	73.5	62.2
Gap (%)	7.7*	3.6	4.6	3.6	5.1	3.8	7.6**	5.0*
Ratio	1.2	1.1	1.1	1.1	1.1	1.1	1.1	1.1
Availability of drugs								
Above the median (%)	50.6	58.6	68.3	72.2	76.0	74.6	79.3	68.5
Below the median (%)	49.7	41.8	61.0	62.9	73.2	64.6	76.0	61.5
Gap (%)	0.9	16.8***	7.3*	9.3**	2.8	10.0**	3.3	7.0***
Ratio	1.0	1.4	1.1	1.1	1.0	1.2	1.0	1.1

Analysis restricted to intervention facilities only (*n* = 75); Gap is the difference in payout score between two subgroups of facilities; ratio is the ratio of payout scores for two subgroups; the significance test was by *t*-test for the null hypothesis of gap equals zero; the results were generally similar when non-parametric test (Wilcoxon rank-sum) was used to test the significant of the gap (results not shown).

* Significance at 10% level.

** Significance at 5% level.

*** Significance at 1% level.

### Heterogeneity in service coverage outcomes

Baseline facility-based delivery rates and coverage of IPT2 during ANC were similar between most facility subgroups (Table 5). Exceptions were higher facility-based delivery rates in facilities with the least poor catchment populations, and higher coverage of IPT2 among the poorest catchment populations. Coverage of IPT2 was higher among dispensaries than health centres and hospitals, but there were lower levels of coverage in both outcomes in the comparison arm at baseline (Table 5).

**Table 5.** Baseline coverage levels by facility subgroups across study arms

Outcome variable/subgrouping variable	Intervention arm (*n* = 75)			Comparison arm (*n* = 75)		
	Yes	No	Gap	Yes	No	Gap
	(1)	(2)	(3)	(4)	(5)	(6)
**OUTCOME 1: institutional/facility-based deliveries**						
Public facility (%)	84.6	84.7	–0.1	86.4	89.0	–2.6
Dispensary facility (%)	82.5	89.5	–7.0	85.3	90.7	–5.4**
Facility with utilities (electricity and water supply) (%)	86.9	81.7	5.2	88.3	85.4	2.9
Facility with drugs availability below the median (%)	83.9	85.2	–1.3	88.6	85.1	3.5
Facility with poorest catchment population (%)	84.6	89.7	–5.1*	81.5	92.7	–11.2***
Facility with middle wealth catchment population (%)	79.5	89.7	–10.2**	86.3	92.7	–6.4**
Facility in rural district (%)	83.9	87.1	–3.2	85.9	92.0	–6.1*
Lower performer (below the median) (%)	73.9	95.6	–21.7***	80.4	95.9	–15.5***
**OUTCOME 2: provision of IPT2 to ANC clients**						
Public facility (%)	50.2	50.6	–0.4	57.0	51.3	5.7
Dispensary facility (%)	53.8	41.7	12.1***	54.1	60.5	–6.4*
Facility with utilities (electricity and water supply) (%)	47.7	53.2	–5.5	57.8	54.1	3.7
Facility with drugs availability below the median (%)	53.6	46.7	6.9*	57.2	54.7	2.5
Facility with poorest catchment population (%)	49.5	45.7	3.8	61.6	52.5	9.1**
Facility with middle wealth catchment population (%)	55.5	45.7	9.8**	53.8	52.5	1.3
Facility in rural district (%)	50.8	47.9	2.9	56.1	55.3	0.8
Lower performer (below the median) (%)	37.3	63.5	–26.2***	44.0	68.9	–24.9***

We used a *t*-test to test the null hypothesis of a gap (Columns 3 and 6) equals to zero; Terciles classified in each arm separately were used for wealth status of the facility’s catchment population; availability of drugs included 37 essential drugs and analysis used a dummy variable classified in each arm separately based on baseline availability distribution (=1 for availability below the median/bottom half and 0, otherwise); reference category for ‘NO’ column in brackets: public (vs non-public), dispensary (vs health centre and hospital), with electricity and water supply at baseline (vs none), baseline availability of drugs below the median/in bottom half (vs top half), baseline lower performer/below the median (vs higher performer); similar pattern of results when hospitals are excluded for facility-based delivery outcome; overall baseline coverage in facility-based deliveries was (84.7 and 86.8%) and IPT2 coverage was (49.5 and 56.7%) for intervention and control arm, respectively (Binyaruka *et al.*, 2015).

* Significance at 10% level.

** Significance 5% level.

*** Significance at 1% level.

P4P resulted in a greater increase in facility-based deliveries among facilities with lower baseline coverage levels than those with higher baseline coverage levels (by 13.0% points, *P* = 0.006) (Table 6), and among facilities serving middle wealth populations than those serving least poor populations (by 14.3% points, *P* = 0.004) (Table 6). P4P also resulted in a greater increase in facility-based deliveries among facilities in rural compared with urban districts (by 10.0% points, *P* = 0.030). The effect of P4P on coverage of IPT2 increased over time and was similar across all facility subgroups (Table 6).

**Table 6.** Heterogeneity in the effect of P4P on service coverage outcomes

	(1)	(2)	(3)	(4)	(5)	(6)	(7)
**Outcome 1: facility-based delivery**							
P4P effect	4.0	5.7	9.2***	4.4	1.0	1.4	–0.8
P4P effect×public facility	4.4						
P4P effect×dispensary facility		2.7					
P4P effect×with available utilities			–2.9				
P4P effect×low availability of drugs				6.3			
P4P effect×lower baseline performer					13.0***		
P4P effect×poorest population						4.0	
P4P effect×middle wealth population						14.3***	
P4P effect×rural facilities							10.0**
Control mean at baseline	86.8	86.6	86.8	86.4	86.5	86.5	86.8
Observation (*n*)	300	300	300	300	300	300	300
**Outcome 2: IPT2 coverage**							
P4P effect	5.4	15.9***	9.4*	10.2**	5.8*	9.2*	4.8
P4P effect×public facility	4.5						
P4P effect×dispensary facility		–9.6					
P4P effect×with available utilities			–0.2				
P4P effect×low availability of drugs				–1.8			
P4P effect×lower baseline performer					7.5		
P4P effect×poorest population						6.4	
P4P effect×middle wealth population						–6.4	
P4P effect×rural facilities							5.2
Control mean at baseline	51.4	51.2	51.6	51.6	51.4	51.9	51.7
Observation (*n*)	300	300	300	300	300	300	300

All regressions are ordinary least square (OLS). All specifications leads to an estimated Beta showing percentage point after controlling for a year dummy, facility-fixed effects and facility-level covariates (availability of utilities and wealth status of the catchment population); availability of drugs include 37 drugs and analysis used a dummy variable classified in each arm separately based on baseline availability distribution (=1 for availability below the median/bottom half and 0, otherwise); reference category in brackets: public (vs non-public), dispensary (vs health centre and hospital), with electricity and water supply at baseline (vs none), baseline availability of drugs below the median/in bottom half (vs top half), baseline lower performer/below the median (vs higher performer), rural (vs urban district), poorest/middle wealth (vs least poor).

* Significance at 10% level.

** Significance 5% level.

*** Significance at 1% level.

The results on facility-based deliveries were similar when we restricted the analysis to primary care facilities, except for the difference between rural/urban locations that became insignificant (Supplementary Table S3). The results were generally robust to clustering at the district level, except that there was no longer a differential effect on deliveries by wealth subgroups (Supplementary Table S4). When two quantiles of wealth scores (lower and higher) were used, the differential effect for facility-based deliveries became insignificant (Supplementary Table S5). The use of non-parametric tests of differences between payouts across facilities revealed similar results to those using parametric tests (Supplementary Table S6).

## Discussion

We examined the distribution of P4P payouts over time and assessed how P4P effects on service coverage differed across facility subgroups in Tanzania. We then assessed whether facility performance was shaped by the incentive design and/or facility and area-based characteristics. This study is one of the few that examine how P4P payouts are distributed and that examine broadly whether there was supply-side heterogeneous P4P effects due to incentive design or structural factors in a LMIC. We found some evidence of both incentive design effects, and effects from structural differences at baseline on performance inequalities. However, the inequalities in payouts distribution declined over time.

Our finding of reduced inequalities in payouts distribution (convergence in performance) by population wealth status over time is partly consistent with the ‘inverse equity hypothesis’ (Victora *et al.*, 2000). The hypothesis suggests that better-off groups will initially benefit from a new intervention, widening inequalities, but over time the worse-off will catch up especially when the better-off have extracted maximum benefit. This convergence in payouts over time is also consistent with US evidence that wealthier hospitals initially received higher payouts than their counterparts, but the distribution of payouts levelled over time (Ryan *et al.*, 2012). The reduced payout inequalities in the US was partly due a change in the incentive design from only rewarding top performers to rewarding any improvement where all providers were likely to receive a payout (Ryan *et al.*, 2012).

The finding that P4P had greatest effect on facility-based deliveries (with multiple threshold targets) among baseline lower performers indicates convergence in performance and is consistent with evidence on quality improvements from the UK (Doran *et al.*, 2008), Canada (Li *et al.*, 2014) and the US (Rosenthal *et al.*, 2005; Lindenauer *et al.*, 2007; Blustein *et al.*, 2010; Chen *et al.*, 2010; Jha *et al.*, 2012). In Rwanda, however, a P4P programme rewards on a fee-for-service system and several rewarded services improved most among facilities with middle baseline quality scores (Sherry *et al.*, 2017). The convergence in performance in HICs was partly linked to a design with multiple threshold targets in the UK (Doran *et al.*, 2008) and Canada (Li *et al.*, 2014) and to a US design system (relative incentive design) that rewarded the highest performers and penalized the lowest performers (Rosenthal *et al.*, 2005; Lindenauer *et al.*, 2007). However, another study in the UK of a hospital incentive scheme with multiple thresholds found evidence of divergence in performance in relation to mortality outcomes linked to pneumonia but not for other conditions (Sutton *et al.*, 2012).

Our finding that the effects of P4P on facility-based deliveries differed according to the wealth status of facility catchment populations is somewhat different to that reported in the UK and the US with respect to quality of care improvements (Doran *et al.*, 2008; Gravelle *et al.*, 2008; Alshamsan *et al.*, 2010; Blustein *et al.*, 2010; Chien *et al.*, 2012; Kontopantelis *et al.*, 2013). While these studies found that providers serving low-income populations performed initially less well but improved most over time, we found that facilities serving middle wealth populations with initial low coverage improved more over time than those with least poor populations. Moreover, while we found that the effect of P4P on coverage of institutional deliveries was greater for rural facilities in Tanzania, a US study found no association between performance on quality and rural/urban location (Ryan and Blustein, 2011); and studies in the UK showed that P4P had less effect in rural than in urban areas (Gravelle *et al.*, 2008; Kontopantelis *et al.*, 2013).

We found similar improvements on IPT2 coverage across facilities (no differential effect of P4P), which is in contrast to literature that suggests a design with a single threshold target, as used for IPT2, fails to account for baseline performance and can enhance divergence in performance (Heath *et al.*, 1999; Rosenthal *et al.*, 2005; Rosenthal and Dudley, 2007; Mehrotra *et al.*, 2010; Mullen *et al.*, 2010; Eijkenaar, 2013). Our finding might be explained by the almost universal coverage of one ANC visit in Tanzania (Binyaruka *et al.*, 2015; TDHS, 2016), and the nature of the targeted service (content of care, rather than service use) may have meant that minimal effort was needed for providers to achieve the target for IPT2.

Our results lend support to the notion that the incentive design, facility characteristics and the nature of services being targeted themselves, will determine how providers respond to P4P, their ability to achieve targets and receive P4P payouts, and the extent to which P4P leads to convergence in performance across providers. Although P4P is typically talked about as a single or uniform intervention, there is in fact substantial variation in incentive structures and scheme designs (Eijkenaar, 2013; Miller and Babiarz, 2013). Our study suggests that design details may be important for determining the distributional effects of P4P across providers, and whether P4P will enhance or reduce existing performance inequalities (Rosenthal *et al.*, 2005; Rosenthal and Frank, 2006; Rosenthal and Dudley, 2007; Ryan *et al.*, 2012). Further research is needed to examine the effects of incentive design on outcomes, and researchers should be encouraged to report on programme design aspects in their evaluations of P4P and systematically monitor and report subgroup effects across providers.

In addition to consideration of incentive design, a number of policies could be introduced to tackle structural factors to increase the likelihood of reducing performance inequalities with the introduction of P4P. ‘Equity bonuses’ have been suggested as a means to enhance performance among disadvantaged facilities so they benefit from payouts from the start (Rosenthal and Dudley, 2007; Meessen *et al.*, 2011; Fritsche *et al.*, 2014). Facility readiness assessment studies and potential quality boosting investments are also important to harmonise the capacity to deliver services prior to P4P. These are standard practices for most P4P programmes funded by the World Bank in LMICs, and the national P4P rollout programme in Tanzania has similarly incorporated these practices.

This study has a number of limitations. First, the administrative data on payouts did not allow for a disaggregation of payouts by service indicator, and thus we used the total payout per cycle which reflects performance across all P4P indicators. Second, since information about payout distribution was limited to intervention facilities, our results represent associations rather than causal effects. Third, we used household data from a random sample of 20 households per facility to proxy service coverage at facility level and wealth status of the facility’s catchment population, and these may have not been representative of the entire catchment populations surrounding facilities. Furthermore, our analysis assumed that households in a facility’s catchment population would have used the facility for care seeking, whereas it is possible that households bypassed their nearest provider to seek care at higher level or more distant facilities. Fourth, the finding of the convergence in coverage of institutional deliveries over 13 months may reflect a regression to the mean principle (a random fluctuation rather than a true causal effect) due to a ‘shorter term’ assessment (Barnett *et al.*, 2005), although the distribution in terms of payouts over the ‘longer term’ of seven payment cycles showed a consistent pattern on convergence. Fifth, as our two service coverage outcomes differed both in terms of incentive design as well as the nature of the service being targeted, it was not possible to determine the extent to which the difference in provider performance response was due to the former or the latter. Finally, because of sample size constraints, we examined differential effects across facility subgroups using a three-way interaction term, and were unable to run separate models for each subgroup (subgroup effects) and compare their effects for better understanding of programme effect. We also classified baseline performance into two subgroups rather than five subgroups as used in the design, due to insufficient sample size. As a result, it was not possible to determine what effect the ‘maintain coverage’ target had on performance relative to the ‘improve coverage’ target.

## Conclusion

In this study, better-off facilities (hospitals, health centres, facilities with more medical commodities and serving wealthier populations) benefited more from P4P payouts than worse-off facilities in the short term; but these inequalities declined over time as worse-off facilities caught up. The increased coverage of facility-based deliveries was greater among facilities with lower levels of baseline coverage, with middle wealth catchment populations, and located in rural areas; whereas the increased IPT2 coverage was similar across facility subgroups. The design of incentives and a range of facility characteristics seem to have influenced performance inequalities; however, the nature of the service being targeted is also likely to have affected provider response. While P4P can help to improve service coverage and quality, and to reduce performance inequalities, care must be taken to ensure that P4P design does not disproportionally benefit those who are already better-off.

## Ethical approval

The evaluation study received ethical approval from the Ifakara Health Institute institutional review board (approval number: 1BI1IRB/38) and the ethics committee of the London School of Hygiene & Tropical Medicine. Study participants provided written consent to participate in this study, requiring them to sign a written consent form that was read out to them by the interviewers. This consent form was reviewed and approved by the ethics committees prior to the start of the research.

## Supplementary Material

Supplementary Appendix
