# Supplementary material for: Does payment for performance increase performance inequalities across health providers? A case study of Tanzania
Source: Health Policy Plan. 2018 Oct 31;33(9):1026–36. doi: 10.1093/heapol/czy084 (PMC6263023; doi:10.1093/heapol/czy084)
Supplement: Supplementary Appendix [file czy084_supplementary_appendix.docx]

**APPENDIX**

**Appendix A1: List of 37 essential drugs**

| Category/ type of classification | Number of items | List of medical commodities considered |
| --- | --- | --- |
| Medicines combined | 37 | Antimalarials, antibiotics, antihypertensives, antidiarrheals, oxytocics, ARVs, vaccines, vitamin A, and family planning medicines |
| 1. *Antimalarials* | 3 | Artemether-Lumefantrine (ALU), quinine and Sulfadoxine Pyrimethamine [SP (IPTp)] |
| 1. *Antibiotics* | 6 | Cotrimoxazole, ampicillin, X-Pen injection, gentamycin, flagyl, and chloramphenicol |
| 1. *Antihypertensives* | 5 | Magnesium sulfate, diazepam, aldomet, nifedipine, and hydralazine |
| 1. *Antidiarrheals* | 2 | Oral rehydration salts (ORS) and zinc |
| 1. *Oxytocics* | 3 | Oxytocin, misoprostol, and ergometrine |
| 1. *Antiretroviral therapy (ARTs)* | 7 | Zidovudine, stavudine, lamivudine, lenofavir, nevirapine, efavirenz, and emtricitabine |
| 1. *Vaccines* | 5 | BCG, OPV, DPT, measles and tetanus |
| 1. *Vitamin A* | 1 | Vitamin A |
| 1. *Family planning medicines* | 5 | Contraceptive pills, depo-provera, injectable, IUCD, and implants |

Notes: For measurement, if a commodity was available on the day of the survey, the outcome was coded 1 and 0 otherwise.

**Appendix A2: List of household ownership assets and characteristics for assessing wealth status**

| **No.** | **Variable description** |
| --- | --- |
| 1. | Asset: electricity |
| 2. | Asset: working radio |
| 3. | Asset: working television (TV) |
| 4. | Asset: working DVD |
| 5. | Asset: working mobile phone |
| 6. | Asset: working landline phone |
| 7. | Asset: working iron |
| 8. | Asset: working refrigerator |
| 9. | Asset: working wall watch |
| 10. | Asset: sewing machine |
| 11. | Asset: table |
| 12. | Asset: sofa coach |
| 13. | Asset: cupboard |
| 14. | Asset: motorcycle |
| 15. | Asset: car |
| 16. | Household member with a bank account |
| 17. | Number of sleeping rooms |
| 18. | Source of drinking water: piped water |
| 19. | Source of drinking water: borehole/ covered well |
| 20. | Source of drinking water: open well |
| 21. | Source of drinking water: spring water |
| 22. | Source of drinking water: river/ dam/pond/lake |
| 23. | Toilet type: flush toilet |
| 24. | Toilet type: pit latrine |
| 25. | Toilet type: no/ other toilet |
| 26. | Source of cooking energy: electricity |
| 27. | Source of cooking energy: kerosene/paraffin |
| 28. | Source of cooking energy: charcoal |
| 29. | Source of cooking energy: firewood |
| 30. | Source of light: electricity |
| 31. | Source of light: solar |
| 32. | Source of light: kerosene/ paraffin |
| 33. | Source of light: candle/ firewood |
| 34. | Source of light: torch or other source |
| 35. | Floor material: sand/earth/dung |
| 36. | Floor material: cement |
| 37. | Floor material: other |
| 38. | Wall material: grass/poles/mud wall |
| 39. | Wall material: bamboo with mud wall |
| 40. | Wall material: sundried/ burnt bricks |
| 41. | Wall material: cement blocks |
| 42. | Wall material: stones with mud |

**Table A3: Differential effects of P4P on institutional deliveries – (robustness check by excluding hospitals)**

|  | All facilities  (N=300) | |  | Excluding all hospitals  (N=276) | |
| --- | --- | --- | --- | --- | --- |
|  | No covariates | Facility  covariates |  | No covariates | Facility  covariates |
| Characteristics | Beta† | Beta† |  | Beta† | Beta† |
|  | (1) | (2) |  | (3) | (4) |
| Panel A: Facility-based characteristics |  |  |  |  |  |
| Ownership (=1 for public) | 2.8 | 4.4 |  | 0.5 | 3.3 |
| level of care (=1 for dispensary) | 3.8 | 2.7 |  | 2.1 | 1.8 |
| Availability of utilities (=1 available electricity & water) | –3.8 | –2.9 |  | –1.6 | –1.3 |
| Availability of drugs (=1 below the median) | 6.5 | 6.3 |  | 4.5 | 4.8 |
| Drugs specific (Oxytocics availability) | 1.9 | 1.6 |  | 0.8 | 1.1 |
| Baseline coverage level (deliveries) | 11.3*** | 13.0*** |  | 12.7*** | 14.3*** |
|  |  |  |  |  |  |
| Panel B: Area-based characteristics |  |  |  |  |  |
| Wealth status (=1 for lower wealth status; 0 for higher) | 1.2 | 2.6 |  | 1.0 | 2.0 |
| Wealth status (=1 for poorest population) –Tercile 1 | 2.5 | 3.9 |  | 0.7 | 1.9 |
| Wealth status (=1 for middle population) –Tercile 2 | 12.6** | 14.3*** |  | 11.9** | 13.2** |
| Facility location (=1 for rural district) | 6.6 | 10.0** |  | 3.3 | 7.1 |
|  |  |  |  |  |  |
| Adjusted for facility-level covariates (wealth & utilities) | – | YES |  | – | YES |

Notes: 2-quantiles of wealth status (lower vs. higher) were used; Drugs specific (oxytocics) for deliveries were used; The differential effect by population wealth was insignificant when excluding the by-passers in the model which excludes all hospitals; *** denotes significance at 1%, ** at 5%, and * at 10% level.

**Table A4: Differential effects of P4P on coverage outcomes – (robustness check by clustering at the district level)**

|  | Institutional deliveries | |  | Provision of IPT2 | |
| --- | --- | --- | --- | --- | --- |
|  | No covariates | Facility  covariates |  | No covariates | Facility  covariates |
| Characteristics | Beta† | Beta† |  | Beta† | Beta† |
|  | (1) | (2) |  | (3) | (4) |
| Panel A: Facility-based characteristics |  |  |  |  |  |
| Ownership (=1 for public) | 2.8 | 4.4 |  | 4.7 | 4.5 |
| level of care (=1 for dispensary) | 3.8 | 2.7 |  | –7.9 | –9.6 |
| Availability of utilities (=1 available electricity & water) | –3.8 | –2.9 |  | 0.5 | –0.1 |
| Availability of drugs (=1 below the median) | 6.5 | 6.3 |  | –2.1 | –1.8 |
| Drugs specific availability (Oxytocics/Antimalarials) | 1.9 | 1.6 |  | 4.2 | 3.4 |
| Baseline coverage level (deliveries/IPT2) | 11.3* | 13.0* |  | 7.3 | 7.5 |
|  |  |  |  |  |  |
| Panel B: Area-based characteristics |  |  |  |  |  |
| Wealth status (=1 for lower wealth status; 0 for higher) | 1.2 | 2.6 |  | 6.1 | 6.5 |
| Wealth status (=1 for poorest population) –Tercile 1 | 2.5 | 3.9 |  | 6.3 | 6.4 |
| Wealth status (=1 for middle population) –Tercile 2 | 12.6 | 14.3 |  | –6.9 | –6.4 |
| Facility location (=1 for rural district) | 6.6 | 10.0* |  | 4.9 | 5.2 |
|  |  |  |  |  |  |
| Adjusted for facility-level covariates (wealth & utilities) | – | YES |  | – | YES |
| Number of observations (N) | 300 | 300 |  | 300 | 300 |

Notes: Clustering at the district level with BOOTSTRAPPING method and used 400 reps.; 2-quantiles of wealth status (lower vs. higher) were used; Drugs specific (oxytocics/antimalarials) for deliveries and IPT2 respectively were used; *** denotes significance at 1%, ** at 5%, and * at 10% level.

**Table A5: Differential effects of P4P on coverage outcomes – (robustness check with two quantiles of wealth status instead of terciles)**

|  | Institutional deliveries | |  | Provision of IPT2 | |
| --- | --- | --- | --- | --- | --- |
|  | No covariates | Facility  covariates |  | No covariates | Facility  covariates |
| Characteristics | Beta† | Beta† |  | Beta† | Beta† |
| Area-based characteristics |  |  |  |  |  |
| Wealth status (=1 for lower wealth status; 0 for higher) | 1.2 | 2.6 |  | 6.1 | 6.5 |
|  |  |  |  |  |  |
| Wealth status (=1 for poorest population) –Tercile 1 | 2.5 | 4.0 |  | 6.3 | 6.4 |
| Wealth status (=1 for middle population) –Tercile 2 | 12.6** | 14.3*** |  | –6.9 | –6.4 |
|  |  |  |  |  |  |
| Adjusted for facility-level covariates (wealth & utilities) | – | YES |  | – | YES |
| Number of observations (N) | 300 | 300 |  | 300 | 300 |

Notes: 2-quantiles of wealth status (lower vs. higher) for below and above median were used; *** denotes significance at 1%, ** at 5%, and * at 10% level.

**Table A6: Distribution of facility payout scores by wealth status of the catchment populations (n=75)**

**–** Non-parametric test (Wilcoxon rank sum test) in column 4.

| Payment Cycle | All | Area-based wealth status | | Parametric | Non-parametric |
| --- | --- | --- | --- | --- | --- |
|  | Mean [SD] | Higher status | Lower status | Gap (p-value) | Gap (p-value) |
|  | (1) | (2) | (3) | (4) | (5) |
| CYCLE 1 (%) | 50.1 [19.4] | 53.9 | 46.3 | **7.6 (0.089)** | 7.6 (0.127) |
| CYCLE 2 (%) | 50.3 [19.1] | 56.6 | 43.9 | **12.7 (0.003)** | **12.7 (0.003)** |
| CYCLE 3 (%) | 64.6 [18.8] | 69.7 | 59.6 | **10.1 (0.019)** | **10.1 (0.005)** |
| CYCLE 4 (%) | 67.5 [19.5] | 68.5 | 66.5 | 2.0 (0.664) | 2.0 (0.500) |
| CYCLE 5 (%) | 74.5 [18.5] | 75.8 | 73.3 | 2.5 (0.554) | 2.5 (0.728) |
| CYCLE 6 (%) | 69.6 [20.1] | 73.9 | 65.4 | **8.5 (0.063)** | 8.5 (0.103) |
| CYCLE 7 (%) | 77.7 [16.3] | 79.1 | 76.3 | 2.8 (0.468) | 2.8 (0.410) |
| Pooled–all cycles (1–7) (%) | 64.7 [11.7] | 68.2 | 61.8 | **6.4 (0.015)** | **6.4 (0.028)** |

Notes: P-values in column (5) are for testing the null hypothesis of zero gap [column (2) – (3)] using Wilcoxon rank sum (Wilcoxon rank sum test) test between two subgroups of wealth status; P-values in column (4) are from t-test; SD=Standard Deviation; Two subgroups of wealth status were generated with equal-size from intervention arm separately; Gap=Higher status–Lower status; Ratio=Higher status /Lower status.
